# Case Report: Isolated acetabular myeloid sarcoma as the initial presentation of extramedullary blast crisis in chronic myeloid leukemia: a report of two cases and literature review

**DOI:** 10.3389/fonc.2026.1750765

**Published:** 2026-02-27

**Authors:** Cong Wang, Yuanyuan Nie, Qiuju Liu, Yan Jiang, Qiang Guo, Jing Bai, Shanshan Liu, Sujun Gao

**Affiliations:** Department of Hematology, The First Hospital of Jilin University, Changchun, China

**Keywords:** chronic myeloid leukemia, extramedullary blast crisis, gluteal mass, imatinib, myeloid sarcoma

## Abstract

**Background:**

Myeloid sarcoma (MS) as the initial manifestation of chronic myeloid leukemia (CML), while the bone marrow (BM) remains in the chronic phase, is exceedingly rare.

**Case presentation:**

We report two cases of MS initially presented with hip pain and soft tissue masses. Both patients had unremarkable complete blood counts and BM morphology, and initial core needle biopsies were misinterpreted as non-Hodgkin lymphoma. Further cytogenetic and molecular analyses, however, identified the Philadelphia chromosome, *BCR::ABL* rearrangement, and mutations in *TP53*, *KMT2D* and *STAG2*, establishing the diagnosis of MS secondary to CML in extramedullary blast phase. Both patients received tyrosine kinase inhibitors with or without chemotherapy, nevertheless, their disease progressed rapidly, resulting in death within one year.

**Conclusion:**

Through a comprehensive literature review, we identified 33 additional reported cases with the same diagnosis. This disease predominantly affects middle-aged men, with bone and parosteal soft tissues being the most common involved extramedullary site. Such patients may constitute a biologically distinct subgroup and, in some reports, have shown relatively favorable outcomes. However, our cases follow an aggressive clinical course, possibly influenced by high-risk molecular features. Large-scale clinical studies are required to clarify the biological heterogeneity of this subgroup and to optimize therapeutic strategies.

## 1 Introduction

Myeloid sarcoma (MS) is a rare extramedullary hematopoietic neoplasm of myeloid origin. It is a heterogeneous disease that most frequently occurs in association with acute myeloid leukemia (AML), but may also present in isolation or arise as a manifestation of disease progression in myelodysplastic syndromes (MDS) or myeloproliferative neoplasms (MPN) (1). MS as the initial presentation of chronic myeloid leukemia (CML) while the bone marrow (BM) remains in chronic phase (medullary CP) is exceedingly uncommon. Current understanding of this condition is solely based on a limited cohort of 11 cases and scattered case reports (2). As a result, its clinical features, optimal diagnostic strategy, and long-term outcomes remain poorly defined.

Here, we report two cases of CML in the medullary CP that presented with isolated acetabular MS. Both patients were initially misdiagnosed as peripheral T cell lymphoma (PTCL). The diagnostic challenge stemmed from atypical pathological findings, notably the absence of MPO staining and co-expression of T-cell markers in the extramedullary tissue, alongside unremarkable complete blood count (CBC) and BM morphology. Although tyrosine kinase inhibitor (TKI) treatment was administered promptly, both patients had an aggressive clinical course and died within one year. Prompted by these diagnostic and therapeutic challenges, we present these cases alongside a systematic literature review to delineate the clinicopathological spectrum and management of this rare condition, aiming to facilitate its timely identification by surgeons, pathologists, and hematologists.

## 2 Case presentation

### 2.1 Case 1

**Figure 1:**
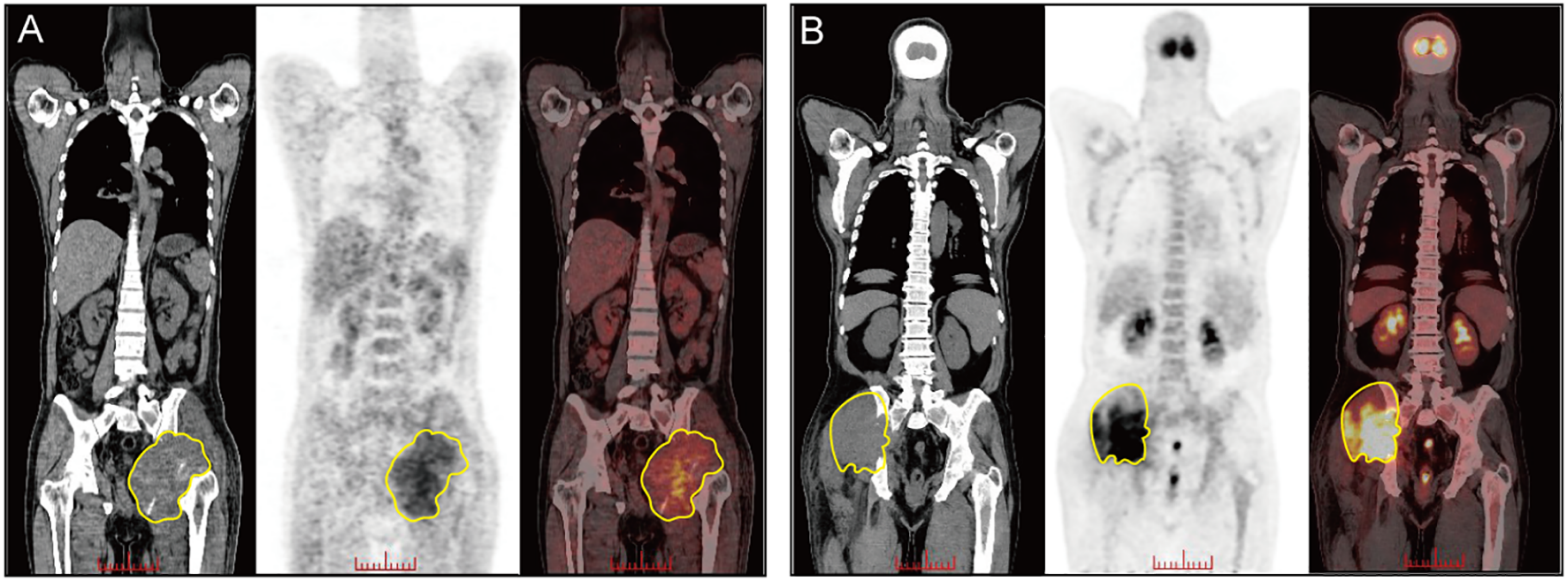
Positron emission tomography–computed tomography (PET-CT) images showing hypermetabolic osteolytic lesions with associated soft tissue masses: **(A)** Case 1, involving the left ischium and acetabulum; **(B)** Case 2, involving the right sacrum and adjacent pelvic bones.

**Figure 2:**
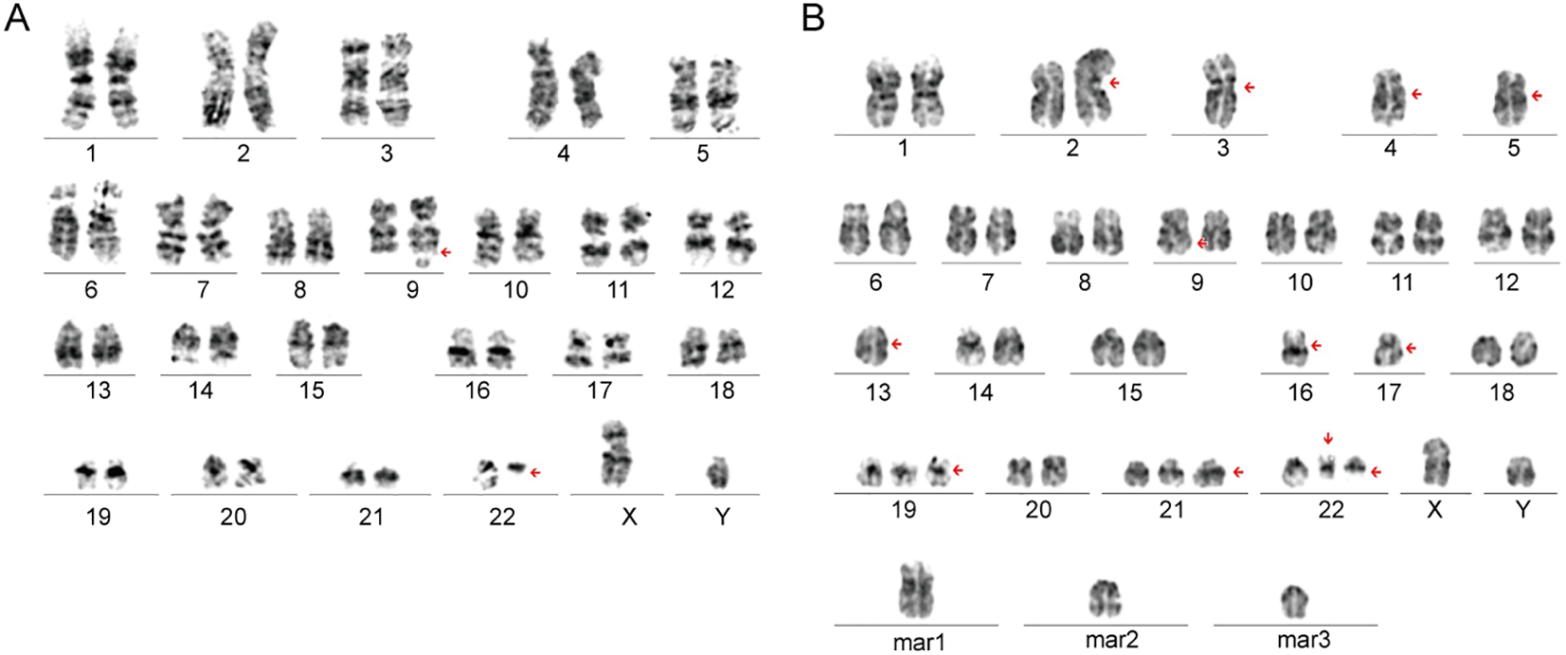
Chromosomal analysis of bone marrow aspirates reveals an evolving abnormal karyotype in Case 1: **(A)** At initial diagnosis: 46,XY,t(9;22)(q34;q11.2)[17]/46,XY[3]; **(B)** At disease progression:46,XY,i(2)(q10),-3,-4,-5,t(9;22)(q34;q11.2),-13,-16,-17,+19,+21,+der(22)t (9;22),+3 mar[3]/46,XY[8].

A 44-year-old male presented with a four-month history of pain in the left gluteal region. Physical examination showed a firm mass in the left hip, accompanied with swelling of the left leg. Position emission tomography-computed tomography (PET-CT) scan (**Figure 1A**) revealed osteolytic destruction of the left ischium and acetabulum, along with an irregular pelvic mass (14.9cm* 12.5cm *9.6cm). Core needle biopsy of the soft tissue lesion revealed a neoplastic proliferation of round to oval cells with a high nuclear-to-cytoplasmic ratio and scanty cytoplasm. Immunohistochemistry (IHC) staining was positive for CD45 and negative for markers of epithelial, mesenchymal and neuroendocrinal origin. Additional immunophenotyping revealed CD117 (+++), CD34 (focal vascular +), CD4 (++), CD7 (local +) and CD3(-), CD2 (-), CD5 (-), CD8 (-), CD10 (-), CD20 (-), CD79a (-), CD1a (-), MPO (-), CD56(-), CD68 (-/+), CD163 (-/+), TdT (-), C-myc (80%), lysozyme (-), Ki-67 (50%). Based on this profile, a provisional diagnosis of PTCL was considered. Further pathological consultations revealed CD33 (+), CD163 (focal +), CD68 (focal +), CD14 (focal +), and CD15(-), supporting a myeloid derived neoplasm with monocytic differentiation. Peripheral blood analysis at presentation revealed a mild leukocytosis (WBC 14,260/μL, normal range 4,000-10,000/μL) and thrombocytosis (platelets 354,000/μL, normal range 100,000-300,000/μL). The differential count was notable for a mild absolute basophilia (90/μL, normal range 0-60/μL), although the relative proportion of basophils remained within the normal range. BM morphology appeared unremarkable. Flow cytometry revealed marked granulocytic hyperplasia (70.62% of nucleated cells) with a normal maturation phenotype, and a minor population of phenotypically normal myeloblasts (0.83%). Comprehensive analysis of all other lineages revealed no immunophenotypic aberrancies. These findings were interpreted as reactive marrow changes without definitive evidence of involvement by myeloid neoplasm. However, cytogenetic analysis of the BM identified a t(9;22)(q34; q11.2) translocation (**Figure 2A**). Molecular testing confirmed the presence of *BCR-ABL1* (p210) fusion transcript. Next-generation sequencing further revealed two genetic alterations: a pathogenic *TP53* p.R175H hotspot mutation (VAF 13.17%) and a *KMT2D* p.V4642I missense mutation (VAF 45.6%). Fluorescence *in situ* hybridization (FISH) analysis of the extramedullary lesion biopsy confirmed *BCR-ABL1* rearrangement in 95% of interphase cells. Mutation screening of the *BCR-ABL1* tyrosine kinase domain (TKD) was initially negative.

Based on these findings, a final diagnosis of MS secondary to CML in extramedullary blast phase was established. Despite sequential regimens of imatinib (600mg once daily) with decitabine (20mg/m^2^, D1-5), imatinib with idarubicin and cytarabine, and dasatinib (70mg twice daily) with homoharringtonine and cytarabine, the extramedullary mass showed no significant reduction, and the *BCR-ABL1* transcript level remained elevated at 154.03%. After six months of regular treatment, the patient developed pancytopenia (neutrophil 300/ul, hemoglobulin 62g/L, platelet 25,000/ul). BM analysis showed marked hypercellularity with 14% myeloblast in both the BM and peripheral blood (PB). Flow cytometry identified an aberrant myeloblast population (16.02%) with an immature, monocytic-biased myeloid immunophenotype: positive for CD33, CD13, CD24, and CD36, partially positive for HLA-DR, and negative for CD34, CD117, CD38, as well as granulocytic maturation markers (CD11b, CD11c, CD15, CD16), confirming medullary involvement by an aggressive myeloid neoplasm. Cytogenetic analysis revealed a complex karyotype with trisomy 19 and double Philadelphia chromosomes (**Figure 2B**), both representing major-route additional cytogenetic abnormalities (ACAs) in CML, indicative of a high risk of disease progression. The *BCR-ABL1* p210 transcript level was elevated to 181.91%. Subsequent *TKD* mutation analysis revealed the immergence of a *T315I* mutation. These results indicated disease progression and resistance to a second TKI therapy. A third-generation TKI was recommended, but the patients opted for palliative care at home and died seven months after initial diagnosis.

### 2.2 Case 2

A 61-year-old male presented with a six-month history of a progressively enlarging mass in the right gluteal region, accompanied by right hip pain. Physical examination revealed an egg-sized lesion palpable around the right hip. PET-CT (**Figure 1B**) revealed a hypermetabolic mass (9.7cm*7.2cm) with associated osteolytic destruction of the right sacrum and pelvic bones. Multiple fluorodeoxyglucose-avid enlarged lymph nodes were present along the iliac vessels and in the right pelvic region, suggestive of lymphoma. Core needle biopsy of the mass was performed and the pathological evaluation was as follows: it was positive for CD45, vimentin, CD30, CD43, CD4, CD7, and negative for CK-pan, S-100, EMA, CD3, CD2, CD5, CD8, CD79a, CD10, MPO, CD56, granzyme B, CD68, ALK. Blood analyses showed a normal white blood cell count and marked thrombocytosis (platelets 711,000/μL, normal range 100,000-300,000/μL). The absolute and relative basophil counts were within normal limits. BM aspirate demonstrated trilineage dysplasia and flow cytometry revealed no abnormal cells. Based on these findings, an initial diagnosis of ALK-negative anaplastic large-cell lymphoma was considered. Subsequent pathologic consultation, however, revealed an IHC profile suggestive of myeloid origin: CD33 (+), CD163 (focal +), and CD68(focal+). Meanwhile, cytogenetic analysis of the BM identified a t(9; 22)(q34; q11.2) translocation. Molecular testing further confirmed the presence of a *BCR-ABL1* (p210) fusion transcript and a co-existing loss-of-function nonsense mutation of *STAG2* (p.R1012X VAF: 73.5%). Furthermore, FISH analysis of the extramedullary lesion biopsy confirmed *BCR-ABL1* rearrangement in 29% of cells.

**Figure 3:**
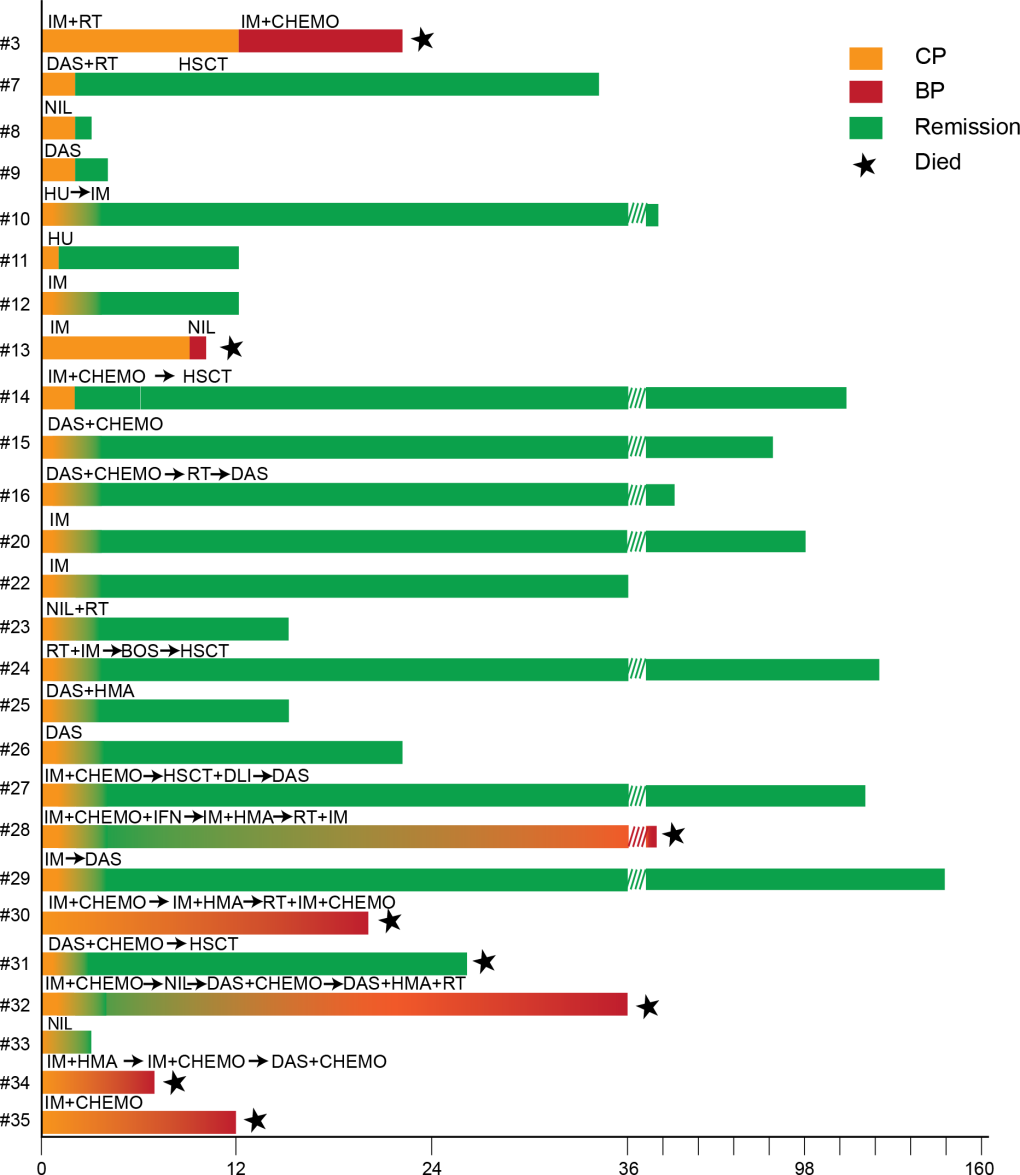
Swimmer plot of treatment courses and outcomes for patients with available survival data from **Table 1**. Bars indicate time on therapy and remission duration, with colors representing different disease phases (Orange: CP, chronic phase; Red: BP, blastic phase; Green: Remission). Asterisk denote death. Abbreviations for treatment: IM, imatinib; RT, radiotherapy; CHEMO, chemotherapy; DAS, dasatinib; HSCT, hematopoietic stem cell transplantation; NIL, nilotinib; HU, hydroxyurea; BOS, bosutinib; DLI, donor lymphocyte infusion; IFN, interferon; HMA, hypomethylating agent.

Together, these findings supported a final diagnosis of MS secondary to chronic myeloid leukemia in extramedullary blast phase. The patient was initiated on imatinib (600mg daily) and transferred to another facility for further management. He subsequently progressed to AML and died approximately one year later. The diagnostic and treatment timelines for these two patients are presented in **Figure 3** and **Table 1**, designated as patients 34 and 35.

**Table 1:** Clinic characteristics of patients with myeloid sarcoma diagnosed in medullary chronic phase of CML.

No	Age	Gender	MS specific involved sites	MS location	WBC (10^9^/l)	Basophil count (10^9^/l)	Basophil (%)	PLT	BM Karyotype	BCR-ABL1 fusion	MS pathology	Treatment	Clinical outcome	OS	Ref
1	62	Female	Pelvis-Acetabulum	Bone and/or Parosteal Soft Tissue	10.4	/	/	331	t(9;22) (q34;q11)	/	CD33+, CD34+, CD43+, CD45+, CD68+, Ki67 80%	/	/	/	Jenkins, et al., 2005 (21)
2	72	Male	Face, Scalp and Trunk	Skin	/	/	/	/	t(9;22) (q34;q11)	/	CD38+, lysozyme+	/	/	/	Watson, et al., 2006 (22)
3	18	Female	Pelvis-Iliac bone	Bone and/or Parosteal Soft Tissue	32.9	/	2	802	t(9;22) (q34;q11)	/	/	Imatinib RT Chemotherapy	Died	22	Torres, et al., 2007 (23)
4	53	Female	Extradural Mass (D10-12)	CNS	220	8.8	6	410	t(9;22) (q34;q11)	/	/	Hydroxyurea Surgery	NA	NA	Chanhan, et al., 2007 (24)
5	53	Male	Chest, Arm, Thigh, Shin	Skin	219	13.1	6	577	t(9;22) (q34;q11)	/	CD13+, CD33+, CD41+, CD61+, CD71+	Imatinib	Remission	/	Nagarajarao, et al., 2009 (25)
6	65	Male	Back	Skin	150	9	6	/	/	/	/	Busulfan	Remission	>6	Wadhwa, et al., 2010 (26)
7	17	Female	Femur	Bone and/or Parosteal Soft Tissue	130.2	20.3	15.6	465	t(9;22) (q34;q11)	P210	CD68(KP-1)+, CD68(PGM-1)+, MPO+	Imatinib, Dasatinib RT HSCT	MMR 4.5	>34	Tsukamoto, et al., 2013 (27)
8	73	Male	Thoracic mass, Parasternal Mediastinal mass, Left-Side Pleural Effusion, LN	Thoracic/Soft Tissue/LN	25	/	/	506	t(9;22) (q34;q11)	/	CD34+, CD117+, CD33+, CD43+, vimentin+	Nilotinib	MMR	>3	Mitchell, et al., 2013 (28)
9	35	Male	Scapula	Bone and/or Parosteal Soft Tissue, LN	18.3	/	/	/	t(9;22) (q34;q11)	P210	CD34+, CD117+, MPO+	Dasatinib HSCT	Remission	>4	Levy, et al., 2014 (29)
10	25	Female	Thigh	Skin	665	/	/	1430	/	/	/	Hydroxyurea Imatinib	Remission	>42	Kumar, et al., 2013 (30)
11	11	Male	Trunk and Extremities	Skin	443	/	/	214	t(9;22) (q34;q11)	/	(FNA) showed immature myeloid cells (BCR/ABL PCR positive)	Hydroxyurea	CHR	>12	Wang, et al., 2014 (31)
12	29	Male	Penile foreskin	Skin	366	21.9	6	622	t(9;22) (q34;q11)	/	LCA+, CD15+, CD34+	Imatinib	CHR	>12	Afrose, et al., 2015 (32)
13	38	Female	Scalp	Skin	146	/	/	417	t(9;22) (q34;q11)	/	MPO+	Imatinib, Nilotinib	Died	9	Sahu, et al., 2016 (33)
14	23	Female	Inguinal LNs	LN	Normal	/	0	/	t(9;22) (q34;q11.2)	P190	MPO+, CD5+, CD7+, CD10+, CD34+, CD43+, TdT+	Imatinib, Dasatinib Chemotherapy HSCT	CMR	>113	Ai, et al., 2015 (34)
15	55	Male	Colon polyp	Intestine	221	/	/	366	t(9;22) (q34;q11)	/	MPO+, lysozyme+, CD43+, CD15+, CD33+, CD34+, CD117+	Chemotherapy dasatinib PKIs	CCyR	>60	Rogers, et al., 2016 (35)
16	29	Male	Brain Parenchyma and Meninges, LN	CNS, LN	34.3	/	0	853	49,XY,+8,t(9;22)(q34;q11.2),+14,del(16q),+21	/	LN: CD45+, CD34+, CD68+, CD38+, CD43+	Dasatinib, Chemotherapy RT	CMR	>48	Abuelgasim, et al., 2016 (36)
17	30	Female	Elbow	Bone and/or Parosteal Soft Tissue	>150	/	/	/	t(9;22) (q34;q11)	/	/	Imatinib	CHR	>3	Mjali et al, 2017 (41)
18	72	Male	Posterior Temporo-Parietal region	CNS	235	7	3	476	t(9;22) (q34;q11)	/	CD45+, CD15+, CD117+(scattered)	Imatinib Surgery	CHR	/	Kabadi, et al., 2017 (37)
19	16	Male	Femur	Bone and/or Parosteal Soft Tissue	461	23	5	443	/	/	MPO+	Imatinib	CHR	>6	Lee, et al., 2020 (14)
20	51	Female	Calf and Forearm	Skin	90.4	/	/	240	t(9;22) (q34;q11)	P210	MPO+, CD34+, lysozyme+, ki67 60%	Imatinib	CHR	>96	Qi, et al., 2021 (38)
21	24	Male	Vertebral body	Bone and/or Parosteal Soft Tissue	102	/	/	540	t(9;22) (q34;q11)	/	LCA+, CD43+, C-Kit (CD117)+, CD68+, ki-67 60–70%	Imatinib Surgery RT	/	/	Shah, et al., 2021 (39)
22	70	Female	Small Intestine	Intestine	23.7	/	/	960	t(9;22) (q34;q11)	/	CD68 (KP-1)+, vimentin+, CD163 (diffusely)+, CD68 (PG-M1)+, CD33+, lysozyme+	Imatinib	Remission	>36	Minato, et al., 2022 (40)
23	73	Male	Paraspinal mass	Bone and/or Parosteal Soft Tissue	/	/	/	/	46,XY,t(9;22)(q34;q11.2)[5]/48,sl,+8,+21[4]/46,XY[11]	/	CD43+, CD45+, CD68+, Vimentin+	Nilotinib RT	MMR	>15	Chen, et al., 2015 (2)
24	51	Male	Bowels/abdominal LNs	Intestine/LN	/	/	/	/	46,XY,t(3;15)(q27;q15),t(9;22)(q34;q11.2)[20]	/	CD45+, MPO+, CD3-, CD20-, CD34-	Imatinib, Bosutinib RT Allo-HSCT	MMR	>126	Chen, et al., 2015 (2)
25	52	Male	Epidural mass (L2-S1)	CNS	/	/	/	/	46,XY,t(9;22)(q34;q11.2)[19]/47,sl,+mar[1]	/	CD45+, MPO+, CD3-, CD20-, CD34-	Dasatinib Decitabine	MMR	>15	Chen, et al., 2015 (2)
26	31	Male	/	Skin	/	/	/	/	t(9;22)(q34;q11.2)	/	MPO+, lysozyme+, CD68+, CD163+, CD117+, CD34+, CD43+	Dasatinib	CCR	>22	Chen, et al., 2015 (2)
27	23	Female	Inguinal LNs	LN	/	/	/	/	t(9;22)(q34;q11.2)	/	MPO+, CD5+, CD7+, CD10+, CD34+, CD43+, TdT+, bcl-2+,	Imatinib, Dasatinib Chemotherapy Allo-HSCT+DLI	CMR	>118	Chen, et al., 2015 (2)
28	47	Male	Arm	Bone and/or Parosteal Soft Tissue	/	/	/	/	t(9;22)(q34;q1 1)	/	CD43+, MPO+, CD3-, LCA (CD45RB), CD20 -	Imatinib Decitabine, troxitabine IFN RT	Died	40	Chen, et al., 2015 (2)
29	50	Male	Cervical LNs	LN	/	/	/	/	46,XY,t(9;22)(q34;q11.2),-18,+der(22)t(9;22)t(18;22)(q11.2;p13)[4]/47,idem,+8[13]/46,XY[3]	/	CD13+, CD33+, chloracetate esterase+, CD7, TdT-, CD10-, CD19-, CD20-, CD79b-, CD5-, CD14-, CD34-	Imatinib, Dasatinib	CMR	>147	Chen, et al., 2015 (2)
30	72	Male	/	Skin	/	/	/	/	46,XY,der(7)t(7;11)(p12;p11.2)add(7)(q35),der(11)del(11)(p11.2)t(11);?(p11.2);?[14]/46,idem,t(9;22)(q34;q11.2)[3]/46,XY[3]	/	D33+, CD68+, CD43+,MPO-, CD117-, CD34-, Lysozyme-	Imatinib Decitabine Chemotheray RT GO	Died	20	Chen, et al., 2015 (2)
31	50	Male	Supraorbital, Pelvis, Bilateral femur	Bone and/or Parosteal Soft Tissue	/	/	/	/	46,XY[20] (*BCR-ABL* FISH positive)	/	CD117+, MPO-, TdT-, CD34-, CD3-, CD5-, CD7-, CD31,	Dasatinib Chemotherapy Allo-HSCT	Died	26	Chen, et al., 2015 (2)
32	54	Male	Femur	Bone and/or Parosteal Soft Tissue	/	/	/	/	t(9;22)(q34;q11.2), +9	/	46,XY,t(9;22)(q34;q11.2)[7]/47,idem,+9[13]	Imatinib, Nitotinib, Dasatinib Chemotherapy Decitabine RT	Died	36	Chen, et al., 2015 (2)
33	63	Female	Pelvis-Sacrum	Bone and/or Parosteal Soft Tissue	/	/	/	/	der(9)t(9;22)(q34;q11.2)del(9)(q34), der(22)t(9;22)	/	46,XX,der(9)t(9;22)(q34;q11.2)del(9)(q34),der(22)t(9;22)[20]	Nilotinib	CHR	>4	Chen, et al., 2015 (2)
34	44	Male	Pelvis-Acetabulum	Bone and/or Parosteal Soft Tissue	14.3	0.09	0.01	354	t(9;22) (q34;q11.2)	P210	Positive for CD33, CD4, CD7, CD117, focally positive for CD163, CD68, CD14,	Imatinib, Dasatinib Chemotherapy	Died	7	
35	61	Male	Pelvis-Sacrum	Bone and/or Parosteal Soft Tissue, LN	4.7	0.01	0	711	t(9;22) (q34;q11.2)	P210	Positive for CD33, CD4, CD7, CD30, focally positive for CD163, CD68, CD14, lysozyme (±)	Imatinib Chemotherapy	Died	12	

## 3 Discussion

MS is a rare malignant tumor of myeloid origin that arises at extramedullary sites, which was recognized as a distinct disease entity in the 2008 World Health Organization classification (3). It represents a heterogeneous group of disorders rather than a single uniform entity. The European Society for Hematology classifies MS into four distinct subgroups according to the associated underlying hematological disease: (1) MS concurrent with AML; (2) extramedullary relapse of AML; (3) blast phase or transformation of MPN, including CML; and (4) *de novo* MS, which occurs in the absence of any concurrent or historical evidence of a myeloid malignancy in the BM or PB (1, 4, 5). The accurate epidemiology of MS is difficult to establish as imaging are not a routine procedure for AML or MPN and that most studies include cases without histological confirmation (6). Kawamoto et al. investigated a large series of MS cases (N = 131) and reported a nearly identical distribution frequency across the four subgroups, with each accounting for approximately one-quarter of the cohort (7). Among the 131 patients, 74% were male, and the median age was 51 years. The most common extramedullary sites were lymph nodes (55%), followed by skin and soft tissues (22.1%), with bone lesions frequently observed in MS following MDS/MPN.

According to the 2022 WHO classification, the diagnostic criteria of MS include effacement of the normal tissue architecture by a mass composed of myeloid blasts, with or without maturing elements, with immunophenotypic evidence of granulocytic and/or monocytic markers. However, establishing a diagnosis of MS in clinical practice remains challenging (6). Approximately 50% of MS cases are initially misdiagnosed as lymphoma, a diagnostic pitfall illustrated in our series. Both patients were initially diagnosed with PTCL mainly based on MPO negativity and aberrant expression of T-cell-associated markers (e.g., CD4 and CD7). The initial diagnostic IHC panels in our cases adhered to a standard, algorithmic approach for undifferentiated hematolymphoid neoplasms. This strategy sequentially evaluates for the most common lineages: B-cell (CD20, CD79a), T-cell (CD3, CD5, CD7), and myeloid (primarily MPO), while also incorporating markers for broader differentials, such as CD56 for NK-cell lineage. The definitive diagnosis of MS was ultimately established by the subsequent positivity for supplementary myelomonocytic markers (CD33, CD68, CD163). As evidenced in our cases and consistent with the literature, the immunophenotype of MS is notably heterogeneous and can be misleading. Myeloid markers exhibit a wide expression range (e.g., MPO in 50~99% of cases, CD33 in 55~94%, CD13 in ~50%, CD15 in 81%, and CD68/KP1 in 60~100%), while aberrant expression of T-cell-associated markers (e.g., CD3 in ~21%, CD5 in ~34%, CD7 in up to 50%) is well-documented (6–9). This immunophenotypic overlap with T-cell lymphomas directly contributed to the initial diagnostic challenge. Therefore, our experience underscores a critical diagnostic rational: when evaluating a poorly differentiated, CD45^+^ neoplasm that lacks canonical lineage-defining markers (MPO, CD3, CD20) yet shows aberrant T-cell marker expression, the initial IHC workup should be strategically expanded. Incorporating a broader panel of myeloid markers, including CD33, CD13 and CD15, alongside monocytic markers (CD68, CD163) is crucial. This approach facilitates earlier detection of MS, particularly in MPO-negative or monocytic variants. Once MS is diagnosed, comprehensive BM evaluation is necessary, even in patients with unremarkable CBC, since the majority of MS are associated with intramedullary diseases and so-called isolated MS often progressed to overt leukemia sooner of latter (8). In our two cases, both patients presented with nearly normal complete blood counts and bone marrow findings that were not classic for chronic-phase CML (a normocellular marrow in Case 1 and dysplastic features in Case 2). The diagnosis would not have been established in a timely manner if not for the detection of Philadelphia chromosome and *BCR-ABL* rearrangement, which ultimately confirmed the disease and highlighted the importance of integrating molecular testing into the diagnostic workflow. Thus, a comprehensive diagnostic approach-including an extensive IHC panel covering myelomonocytic markers, coupled with BM evaluation incorporating karyotyping, FISH and molecular analyses is essential for the accurate diagnosis and subclassifying of MS.

As discussed above, some MS cases arise in the context of MPN, including CML, particularly during the accelerated or blast phase (BP). In such scenarios, MS is recognized as an extramedullary blast crisis of CML, accounting for 7–17% of all CML-BP cases (10). Extramedullary blast crisis typically occurs later in the disease course as a sign of disease progression. Very rarely, MS may present as the initial manifestation of CML, even when the BM and PB still show features of chronic phase, as demonstrated in our two cases. To better understand the clinicopathological, cytogenetic and prognostic features of this rare condition, we conducted a comprehensive literature review. We systematically searched PubMed for articles published between 2000 and 2025 using the query: (“CML” OR “chronic myeloid leukemia”) AND (“myeloid sarcoma” OR “extramedullary”). This was followed by a manual review of the references of retrieved articles. Cases were included only if MS was the presenting feature alongside confirmed medullary chronic-phase morphology (<10% blasts). This process identified 33 reported cases over the past 25 years in which MS was the initial presentation of CML in medullary CP (2). The clinical characteristic of these cases, along with our two additional cases, are summarized in **Table 1**. The median age at MS diagnosis was 50 years (range:11–73). Consistent with the general MS cohort, a male predominance was also observed in this subtype (23/35, male-to-female ratio 1.92:1). The most frequently involved sites were bone and/or parosteal soft tissue (14/35), particularly the pelvic region-including the acetabulum, sacrum, and iliac bone-followed by skin (10/35), lymph nodes (5/35), and central nervous system (CNS) (4/35). Both of our cases involved the pelvic bone and adjacent soft tissue, suggesting extramedullary MS lesions may preferentially originate from the BM of hematopoietically active, irregular bones containing malignant CML cells. Furthermore, peripheral CBC test at diagnosis were available for 23 of the 33 reported cases, the great majority exhibited proliferative features of CML, with leukocytosis (21/23) and thrombocytosis (17/23) being frequently observed. Therefore, for isolated neoplasms in the pelvic region, particularly those accompanied by abnormal CBC findings, hematologists, as well as surgeons and pathologists who may participate in the diagnostic process, should maintain a high index of suspicion for MS. In patients with normal blood counts, especially when the BM is in the chronic phase, morphological assessment alone is often insufficient to conclusively diagnose and classify MS, a challenge illustrated by our two cases. In such scenarios, comprehensive molecular profiling of the BM is essential for an accurate diagnosis.

Regarding further BM examinations for CML, only 25% cases (8/32) exhibited ACAs at initial diagnosis besides the Philadelphia chromosome. Four of these cases were found to have high risk ACAs (trisomy 8, trisomy 18, +Ph, and complex karyotype) (11). This contrasts with the reported ACA frequency of 74.14% in general CML-BP cohorts (12), where double Ph (44.18%) and trisomy 8 (25.58%) are the most common abnormalities. These data suggest that CML patients presenting with extramedullary BP while in medullary CP may have a lower baseline burden of ACAs compared to those with medullary BP. This may indicate that extramedullary BP is driven by alterative mechanisms, such as specific driver mutations or tissue-specific microenvironments, without requiring the full cytogenetic complexity typically associated with medullary transformation. Among 7 cases with available fusion gene data, 71% were positive for the p210 transcript (5/7). In both of our cases, the patients exhibited only the Ph chromosome at initial diagnosis and were positive for the p210 transcript. However, one of them developed a complex karyotype during the medullary blast phase following multiple lines of treatment, suggesting that genomic instability may have contributed to the rapid disease progression. Regarding the pathological diagnosis of MS, among the 28 patients with available IHC data, the most frequently expressed myeloid marker was MPO (13/28), followed by CD68 (11/28) and CD33 (10/28). Therefore, in patients with suspected hematolymphoid neoplasms, the initial IHC panel should include, at a minimum, MPO, CD68, and CD33, in conjunction with other lineage-specific markers, to facilitate accurate diagnosis.

The treatment and prognosis of MS vary significantly depending on the underlying associated disease. In the study by Kawamoto et al., one-year survival rates for *de novo* MS, MS with concomitant AML, and MS following MDS/MPN were 60.0%, 50.1%, and 14.3%, respectively. In that study, patients with MS following MDS/MPN-who had received prior therapies-exhibited treatment resistance and had the poorest outcomes (7). Furthermore, Chen et al. analyzed 307 CML patients, including 42 with MS, and reported that MS patients that developed later in the treatment course while in medullary CP (N = 17) had a median survival of only 8 months, comparable to the poor outcome of later onset medullary blast crisis (7 months). In contrast, patients presenting with MS during medullary CP (N = 13) had a median survival of 36 months and were more likely to achieve complete remission of MS as well as deeper remission of medullary disease (13). In our retrospectively reviewed cohort (N = 35), all patients had CML initially presenting as MS during the medullary chronic phase. The majority received TKI with or without chemotherapy, with some undergoing local radiotherapy or surgical excision of the mass. Six patients proceeded to hematopoietic stem cell transplantation. Overall, these patients demonstrated favorable responses to treatment and achieved deep remissions (**Figure 3**). These findings suggest that MS as an initial presentation of CML with medullary CP may represent a biologically distinct entity compared with MS with medullary blast crisis arising as a late event and may not necessarily confer an adverse prognosis in the TKI era. Furthermore, MS in the initial stage of CML are less likely to have treatment resistance commonly observed in the late sequela of CML (14). Because of its rarity and the lack of randomized trials, MS presenting as the initial presentation of CML is still categorized as blast phase, and chemotherapy plus TKI followed by consolidation with HSCT is recommended (15, 16). As neither patient in our series had prior TKI exposure, imatinib (600 mg daily) was selected as first-line therapy, which was consistent with standard practice for CML at that time. In Case 1, the presence of a *TP53* mutation prompted the addition of decitabine, based on the reported sensitivity of *TP53*-mutated myeloid neoplasms to hypomethylating agents. Despite this biology-driven combination, both patients exhibited primary resistance and rapidly progressed to medullary blast phase. In retrospect and aligned with current guidelines, the diagnosis of CML-BP should have prompted upfront use of a second- or third-generation TKI to achieve faster disease control and enable timely bridging to allogeneic stem cell transplantation. Earlier TKI escalation and transplant referral might have altered the adverse outcomes observed.

Although MS arising in CML chronic phase has generally been associated with relatively favorable outcomes in the literature, our two cases illustrate that individual responses may differ markedly, particularly in the presence of high-risk genetic lesions. The molecular findings in our cases offer crucial insights into the aggressive biology of CML-associated MS. Specifically, the first patient harbored a canonical hotspot mutation in *TP53* p.R175H (VAF 13.17%), which is recognized as a key driver of blast crisis transformation in CML. This mutation indicates marked genomic instability and confers resistance to therapy (17). This patient failed to respond to TKI therapy and subsequently developed high risk ACAs along with a *T315I* mutation. The low VAF of the *TP53* mutation suggests that it might belong to a subclone that underwent clonal evolution toward a more aggressive phenotype following treatment. Conversely, the high VAF of the concomitant *KMT2D* variant may indicate its presence in an earlier, dominant clone, potentially contributing to a permissive epigenetic landscape for subsequent transformation. In the second case, the loss-of-function *STAG2* mutation (VAF 73.5%) was present at a high allele frequency, indicating its clonal dominance. STAG2, as a core component of the cohesin complex, is essential for maintaining chromatin architecture in hematopoiesis and leukemogenesis. Cohesin gene mutations are generally mutually exclusive and have been reported in approximately 12% of AML, 8% of MDS, and 6% of CML (18). *STAG2* mutations are characteristic of secondary AML, where they occur at a notably higher frequency (up to 14%) compared to *de novo* AML (approximately 4%) (19). Therefore, this *STAG2* mutation might represent a key molecular driver of progression to AML. Beyond the high-risk molecular features detailed above, the anatomical location of the MS lesion itself may harbor prognostic significance. In our literature review of 33 cases, we noted that among the 6 reported fatalities, the MS involved bone or parosteal soft tissue in 4 cases and skin in 2 cases. Notably, in line with this patten, both patients in our present series had MS involving parosteal soft tissue. This anatomical concordance suggests that MS arising in or adjacent to bone may be associated with a more aggressive clinical course. A recent systematic review and meta-analysis encompassing 7241 patients with MS demonstrated a pooled median overall survival of 12.8 months, with skin/soft tissue being the most common sites of involvement (20). Notably, among 112 patients with paired DNA sequencing of MS and bone marrow, 56% exhibited discordant mutational profiles, indicating distinct biological niches and clonal heterogeneity between these compartments. Although paired genomic data at progression were unavailable in our cases, immunophenotypic comparison between the initial extramedullary MS and subsequent medullary AML provides surrogate evidence of clonal evolution. In Case 1, the AML blasts retained a monocytic-biased myeloid immunophenotype but demonstrated complete loss of CD117. This immunophenotypic shift likely reflects clonal selection under therapeutic pressure, underscoring the biological heterogeneity of CML-associated MS.

## 4 Conclusion

In conclusion, we have described two cases of CML presented as MS while in medullary CP, which is exceedingly rare and may represent a biologically distinct entity. Accurate diagnosis relies on thorough pathological evaluation with a comprehensive IHC panel and a detailed assessment of the BM. Although many reported cases achieve relatively favorable outcomes with TKI-based therapy, our findings emphasize that genetic lesions-such as mutations in *TP53* and *STAG2*, along with complex karyotype-may drive aggressive disease behavior. Therefore, integrating molecular profiling into the diagnostic and therapeutic workflows is critical, and future multicenter studies will be necessary to establish risk-adapted treatment strategies for this uncommon yet clinically significant presentation.

## Data Availability

The original contributions presented in the study are included in the article/supplementary material. Further inquiries can be directed to the corresponding author.
